# Probing axons using multi‐compartmental diffusion in multiple sclerosis

**DOI:** 10.1002/acn3.50836

**Published:** 2019-08-13

**Authors:** Francesca Bagnato, Giulia Franco, Hua Li, Enrico Kaden, Fei Ye, Run Fan, Amalie Chen, Daniel C. Alexander, Seth A. Smith, Richard Dortch, Junzhong Xu

**Affiliations:** ^1^ Neuroimaging Unit/Neuroimmunology Division Department of Neurology Vanderbilt University Medical Center Nashville Tennessee; ^2^ Neurology Department VA TN Valley Healthcare System Nashville Tennessee 37215; ^3^ IRCCS Foundation Ca’ Granda Ospedale Maggiore Policlinico Dino Ferrari Center Neuroscience Section Department of Pathophysiology and Transplantation University of Milan Milan Italy; ^4^ Institute of Imaging Science Department of Radiology and Radiological Sciences Vanderbilt University Medical Center Nashville Tennessee; ^5^ Centre for Medical Image Computing Department of Computer Science University College London London United Kingdom; ^6^ Department of Biostatistics Vanderbilt University Medical Center Nashville Tennessee; ^7^ Department of Biomedical Engineering Vanderbilt University Nashville Tennessee

## Abstract

**Objects:**

The diffusion‐based spherical mean technique (SMT) provides a novel model to relate multi‐b‐value diffusion magnetic resonance imaging (MRI) data to features of tissue microstructure. We propose the first clinical application of SMT to image the brain of patients with multiple sclerosis (MS) and investigate clinical feasibility and translation.

**Methods:**

Eighteen MS patients and nine age‐ and sex‐matched healthy controls (HCs) underwent a 3.0 Tesla scan inclusive of clinical sequences and SMT images (isotropic resolution of 2 mm). Axial diffusivity (AD), apparent axonal volume fraction (*V*_ax_), and effective neural diffusivity (*D*_ax_) parametric maps were fitted. Differences in AD,* V*_ax_, and *D*_ax_ between anatomically matched regions reflecting different tissues types were estimated using generalized linear mixed models for binary outcomes.

**Results:**

Differences were seen in all SMT‐derived parameters between chronic black holes (cBHs) and T2‐lesions (*P* ≤ 0.0016), in *V*_ax_ and AD between T2‐lesions and normal appearing white matter (NAWM) (*P* < 0.0001), but not between the NAWM and normal WM in HCs. Inverse correlations were seen between *V*_ax_ and AD in cBHs (*r* = −0.750, *P* = 0.02); in T2‐lesions *D*_ax_ values were associated with *V*_ax_ (*r* = 0.824, *P* < 0.0001) and AD (*r* = 0.570, *P* = 0.014).

**Interpretations:**

SMT‐derived metrics are sensitive to pathological changes and hold potential for clinical application in MS patients.

## Introduction

Axonal injury is a key element in the pathology of multiple sclerosis (MS) and a major determinant of patients’ disability.^1^ Because capturing and characterizing axonal damage in MS patients in vivo is challenging,^2^ there remains a pressing need to identify a magnetic resonance imaging (MRI) biometric indicative of axonal quantity. This biomarker would allow for the detection of microscopic damage before symptoms and irreversible tissue damage appear, potentially optimizing treatment personalization based on an accurate characterization of tissue injury. Lack of this biomarker is an impediment to a responsible design and conduct of phase II and phase III clinical trials on neuroprotection,^3^ ultimately making MS an incurable disease.

Combined MRI‐histopathologic studies provided the demonstration that lesions appearing as hypo‐intense on T1‐weighted spin echo (T1‐w SE) MRI in the absence of corresponding enhancement on postcontrast sequences, namely, chronic black holes (cBHs), represent areas of more advanced disease pathology and axonal loss compared with lesions that show only hyper‐intense signal on T2‐w MRIs.^4^ Although cBHs’ count is used as semiquantitative tool to measure axonal integrity, this count remains poorly specific to the degree of axonal pathology. cBHs’ visibility is also affected by the MR field strength and acquisition parameters. MR spectroscopy (MRS) and the quantification of the neuronal metabolite N‐acetyl aspartate (NAA) has made it possible to infer on axonal damage and secondary loss in MS.^5^ Technical disadvantages such as the long acquisition time and poor image resolution challenge the advancement of MRS to clinical and research utilization.^2^

Diffusion‐based imaging offers an opportunity to overcome the limitations of these two techniques.^6^ In diffusion‐based MRI the signal is sensitive to the random motion of water molecules, which is hindered by barriers like myelin and axons in human brains. Diffusion tensor imaging (DTI) models and measures anisotropic diffusion along multiple directions, yielding measures of axial diffusivity (AD = diffusion parallel to fiber bundles), radial diffusivity (RD = diffusion perpendicular to fiber bundles), mean diffusivity (MD = diffusion across all directions) across all directions, and the derived fractional anisotropy (FA). Although it is recognized that a complex relationship links these metrics with tissue biological changes, RD and AD values are referred as proxy of myelin and axonal quantity, respectively.^7^

A major limitation of DTI is that its associated signals arise from all water compartments within a voxel.^7^ Hence, fluid accumulation typically seen during inflammation or end stage cavitation^8^, ^9^ can significantly contaminate the derived metrics. Several advanced multi‐compartment diffusion models have been developed to isolate and separate the signal contribution deriving from different compartments, thus increasing the ability to detect specific tissue structures. For example, diffusion basis spectrum imaging (DBSI) developed by Wang and collaborators^10^, ^11^, ^12^ detects increased cellularity and water content in MS. The neurite orientation dispersion and density imaging (NODDI) is a simple and practical model‐based diffusion MRI technique for estimating microstructural complexity such as volume fraction and dispersion of dendrites and axons in vivo.^13^, ^14^, ^15^

The Spherical Mean Technique (SMT) provides an alternative model to relate multi‐*b*‐value diffusion MRI data to features of tissue microstructure, recently developed by Kaden and collaborators.^16^, ^17^ SMT removes the orientation dependence of diffusion MRI signals by taking the averages of diffusion signals over all gradient directions for each b shell.^16^, ^17^, ^18^ This not only simplifies data analysis but also significantly improves the signal‐to‐noise ratio (SNR), helping to reduce interscan variations. In contrast to DTI derived metrics, which are sensitive to both fiber orientation distribution and fiber microstructure, SMT derived measures are sensitive to fiber composition only, thus more specific to tissue biology. On the other hand, SMT avoids some of the strong modeling assumptions in NODDI and has less computational demands, while having similar modest acquisition requirements to NODDI (two non‐zero *b*‐values) and much simpler acquisition requirements compared with DBSI.

The SMT model has two compartments, that is, the intra‐axonal and extra‐axonal space. In the intra‐axonal compartment, SMT models (1) the parallel diffusivity and (2) perpendicular diffusivity, which is set to zero because of the very small diameter of the axons; and the (3) apparent volume fraction (*V*_ax_). In the extra‐axonal compartment, SMT models (1) the parallel diffusivity and (2) perpendicular diffusivity, which depends on the packing density of the (myelinated) axons; and the (3) volume fraction 1 − *V*_ax_. Relevant to the studies we propose, SMT estimates, the apparent axonal volume fraction (*V*_ax_) and the effective neural diffusivity (*D*_ax_). *V*_ax_ is a proxy of the (%) signal fraction arising from axons inside a given voxel, hence relating to the apparent volume fraction of occupied by axons within a given voxel. *D*_ax_ averages the parallel diffusivity over the intra‐ and extra‐axonal water pools and provides indirect estimates of microenvironments inside the axons and their surroundings.

Work in our group has validated SMT technique against histology using mice with tuberous sclerosis complex and age‐matched controls.^16^ This animal model is more suitable to detect axonal injury in brain than that of experimental autoimmune encephalomyelitis typically used to study MS, as free from the inflammatory component which can function as confounding factor in a validation process. Animals with tuberous sclerosis complex showed significantly decreased values of *V*_ax_ across several affected brain regions compared with controls. A decrease in the myelinated axon fraction (with the myelin fraction excluded) was mirrored by a reduction of the MRI‐based *V*_ax_.^16^ Although obtained with a different disease model, the provided validation against axonal histology is fundamental and applicable to any condition affecting myelin and axonal integrity.

Given that axonal injury and loss are markers of MS pathology and SMT‐derived indices report on the fraction of viable axons, we hypothesize that indices derived from SMT are sensitive to different degrees of pathological changes and, thus hallmarks of neurodegenerative tissue injury in patients. To test this hypothesis, we acquired SMT in a cohort of MS patients and of age‐ and sex‐matched healthy controls (HCs). We derived *V*_ax_ and *D*_ax_, measurements in tissue with different degree of injury based on gold‐standard conventional clinical MRI with the goal of assessing clinical feasibility and translation for MS patients imaged at a 3.0 Tesla (3T) scanner.

## Methods

### Study design and cohort

This cross‐sectional study was performed as collaborative project between the Neurology and the Radiology Departments at the Vanderbilt University Medical Center (VUMC) and the University College London. The study was approved by the VUMC Institutional Review Board and each subject signed an informed written consent prior to be enrolled. All methods were performed in accordance with the relevant guidelines and regulations.

Eighteen MS patients and nine age and sex‐matched HCs were consecutively included. Patients were required to be ≥6 months free from clinical relapses/steroids administration and to have no disease activity documented by a postcontrast clinical scan within the previous month (Table 1). Exclusion criteria for both patients and HCs were: (1) contraindication for a brain MRI; (2) pregnancy and/or breastfeeding; (3) other brain autoimmune, neoplastic or infectious diseases, current or past ≥6 months longer history of uncontrolled hypertension, diabetes, hyperlipidemia, and migraine; (4) inability to provide a written consent. Each subject underwent a clinical evaluation and a brain MRI scan within 2 ± 2 weeks. In patients, the clinical exam consisted of obtaining an accurate history and rating the disability using the Expanded Disability Status Scale (EDSS)^19^ score and the average of two trials for the Timed 25‐Foot Walk Test (T25‐WT).^20^ For HCs, morbidities were ruled out via a questionnaire. Four subjects were imaged twice within 2 ± 2 weeks to compute interscan variability measures.

**Table 1:** Demographic, clinical, and MRI characteristics of patients

	Patients (*n* = 18)	HCs (*n* = 9)	*P*‐value
Age	45.5 ± 14.6	41.7 ± 10	0.486*
Sex (females/males)	13/6	5/4	0.507**
Ethnicity	2 African American 16 Caucasians	8 Caucasians 1 Asian	–
MS type (CIS/RRMS/SPMS)	4/11/3	–	–
Disease duration (years)	12.1 ± 10.6	–	–
EDSS score	1 (0–6.5)	–	–
T25‐FW (seconds)	10:37 ± 18:12	–	–
T2‐lesions volume (cm^3^)	14.9 ± 21.3	–	–
cBHs volume (cm^3^)	7.3 ± 7.5	–	–

Quantitative data are expressed in mean ± SD (minimum‐maximum value) except for the EDSS score for which median (minimum‐maximum values) are reported; cBHs, chronic black holes; CIS, clinically isolated syndrome; EDSS, expanded disability status scale; RRMS, relapsing remitting multiple sclerosis; SPMS, secondary progressive multiple sclerosis. *P* values generated by *unpaired Student's *t*‐test and a **χ ^2^ test.

### MRI protocol and image postprocessing (Fig. 1)

**Figure 1:**
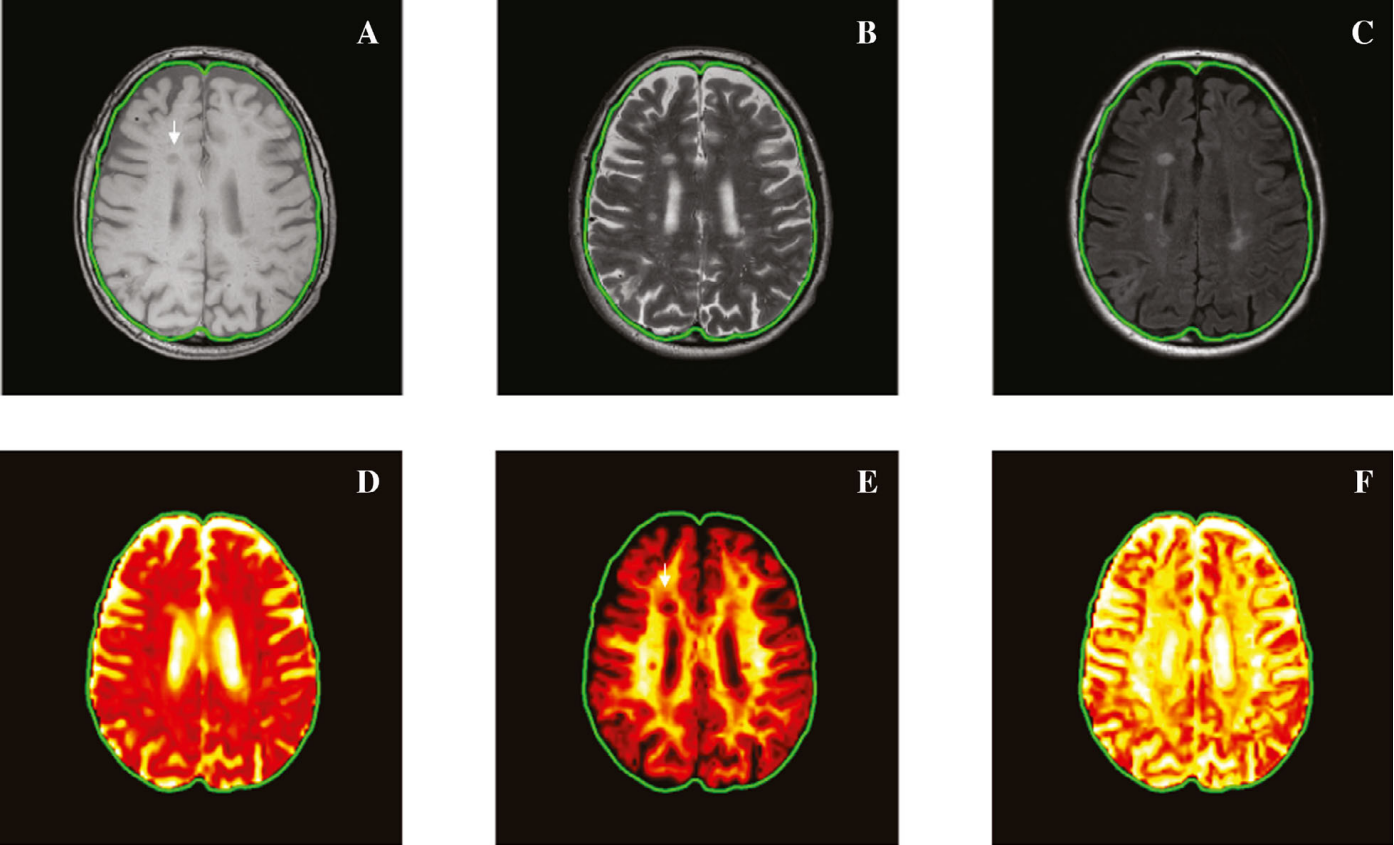
Coregistered anatomical MRI and parametric maps. Top row shows T1‐w TSE (A), T2‐w TSE (B), and T2‐w FLAIR (C); bottom row shows AD (D), *V*_ax_ (E), and *D*_ax_ (F) maps. Note the cBH visibility on T1‐w SE image and *V*_ax_ map (white arrow).

Brain MRIs were performed using a Philips Achieva 3T whole‐body scanner equipped with a 32‐channel receiver head coil. All sequences were acquired without gap. Whole‐brain T1‐w and T2‐w turbo SE (TSE), T2‐w fluid attenuated inversion recovery (FLAIR) images were obtained at a resolution of 0.4 × 0.4 × 2 mm^3^. SMT images were acquired with isotropic resolution of 2 mm. Diffusion MRI was acquired using single‐shot, SE echo planar imaging (EPI) readout, echo time (TE) = 74 msec, repetition time (TR) = 13.5 sec, 2 b shells (1000 and 2500 sec/mm^2^), 90 diffusion directions (45 directions on each *b* shell) according to a previously reported approach.^21^ An additional *b* = 0 image volume was acquired with reversed phase‐encode EPI blips to correct for image distortion.

For each subject, all diffusion MRI data were corrected to remove susceptibility and eddy‐current induced distortion using topup and eddy toolbox in FSL (https://fsl.fmrib.ox.ac.uk/fsl/). Two parametric maps, *V*_ax_ and *D*_ax_ were fitted according to the method reported previously.^17^ In addition, 3D DTI‐based parametric maps AD were generated. Thereafter, T1‐w TSE, T2‐w FLAIR MRIs, and each of the SMT derived parametric maps were coregistered to the T2‐w TSE image using an affine coregistration (flirt toolbox^22^ in FSL, https://fsl.fmrib.ox.ac.uk/fsl/). Figure 1 displays the array of reconstructed maps and registered clinical scans of an MS patient chosen as example.

### Analysis of SMT‐derived metrics across tissue types (Fig. 2)

**Figure 2:**
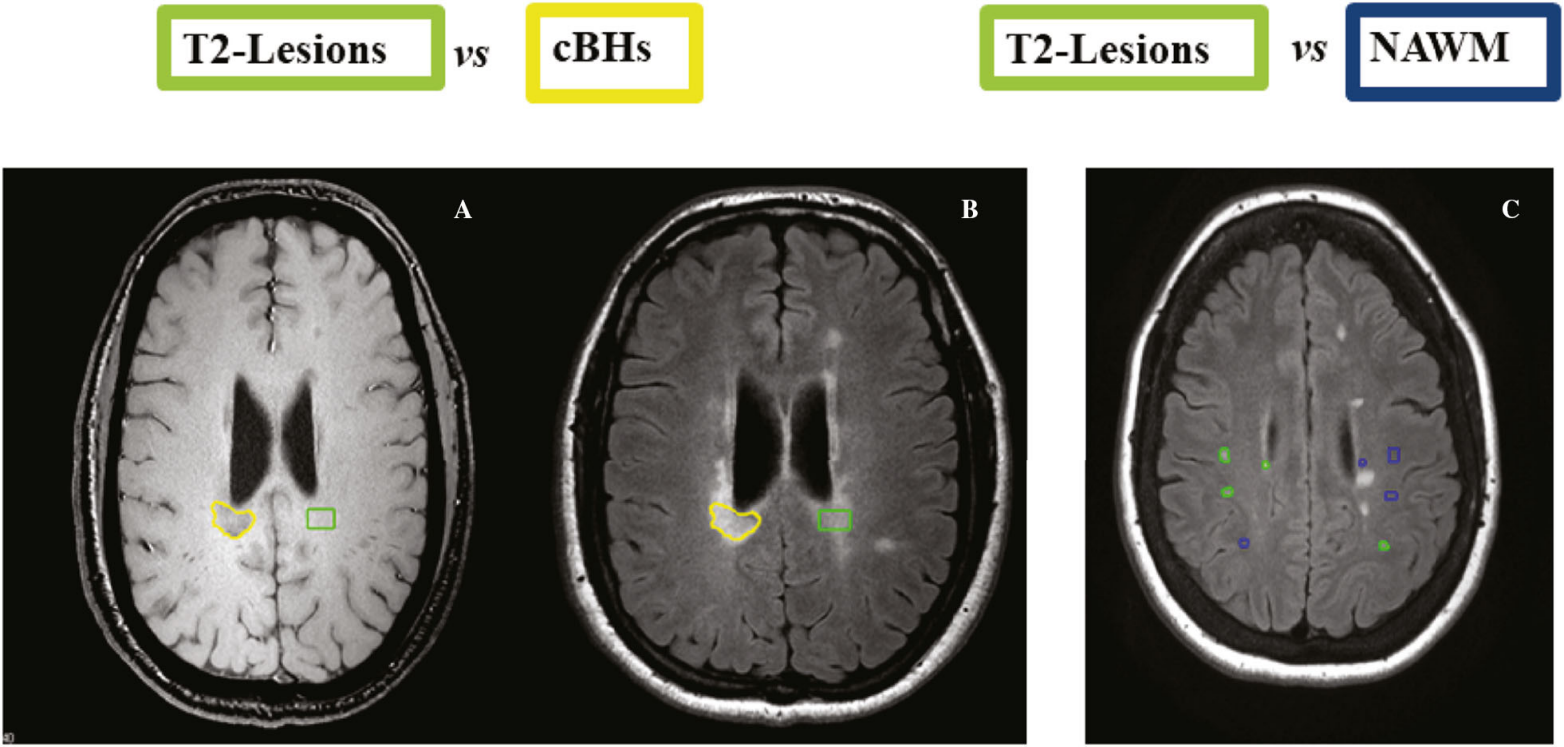
Schematic representation of lesions delineation. Lesion delineation is illustrated. Each cBH (yellow region‐of‐interest (ROI) in A and B) was anatomically matched with a correspondent T2‐lesion (green ROI in A and B); each T2‐lesion (green ROIs in C) was anatomically matched with contralateral areas of normal appearing white matter (NAWM, blue ROIs in C).

To quantify differences between various tissues types, given the well‐known variability in axonal number and diameter in different areas of the brain, anatomically matched regions of interest (ROIs) were generated (Fig. 2). This evaluation was achieved by inspecting T1‐w and T2‐w coregistered images at the same time and ensuring presence/absence of a specific tissue type in the area of interest and in the contralateral one. ROIs (≥10 voxels)^8^ were delineated using graphic tools available in MIPAV (https://mipav.cit.nih.gov). All generated masks were then overlaid on *V*_ax_, *D*_ax_, and AD parametric maps and correspondent quantities derived using mathematical tools in MIPAV.

ROIs were placed on areas of normal appearing white matter (NAWM), T2‐lesions and cBHs in patients, and in normal WM (NWM) in HCs. Tissue type classification was based on visual inspection performed by a junior investigator (GF) and confirmed by a senior investigator (FB).

NAWM was defined as any area displaying normal signal on T2‐w TSE and FLAIR images. Care was taken to avoid areas of dirty WM^23^ and those in the vicinity of a lesion. T2‐lesions were defined as any area of hyperintense signal on T2‐w TSE and FLAIR images^24^ that did not have a corresponding hypo‐intense signal on T1‐w TSE MRI. cBHs were defined as areas of hypo‐intense signal on T1‐w TSE corresponding to a region of hyperintense signal on T2‐w TSE and FLAIR images.^25^ None of the lesions was known to be in active stage given our inclusion criteria.

This tissue type classification was based on the known concepts that: (1) a considerable amount of pathology may be found in areas of MS brains appearing as “normal” on conventional MRI scans^1^; (2) T2‐lesions without a corresponding cBH carry pathological traits that are less severe than cBHs^8^; and (3) cBHs are expected to harbor variable degrees of axonal injury.^4^

To quantify differences between NAWM (patients) and NWM (HCs), ROIs were delineated in the internal capsula only (chosen as representative tract). The internal capsula was localized by two investigators (GF and FB) looking at the T2‐w images against anatomical atlases. For this last set of comparisons, care was taken that all areas had the same size which was 80 voxels.

### Analysis of SMT‐derived metrics and lesion burden

To assess the associations between overall lesion burden and SMT‐derived metrics, whole‐lesion load on T2‐w and T1‐w images was computed. To obtain lesion volume, we used the procedures previously described.^26^ In brief: we first identified and delineated all cBHs on T1‐w TSE MRIs and all hyperintese WM lesions on T2‐w FLAIR. Thereafter we created a subtraction mask in MATLAB (Mathworks, Natick, MA) by removing all the cBHs from the hyperintense lesions. The cBHs masks were used to compute the cBHs volume and the subtraction mask was used to compute the overall T2‐lesion burden, using mathematical tools available in MIPAV.

### Interscan variability measurements

For interscan variability measurements, ROIs were traced on T2‐w SE MRI randomly obtained with the first or the second scan. SMT imaging derived maps at each time point were then registered into the T2‐w SE space selected for ROI labeling using the FSL software package as described above. For each pair of scans, ROIs were selected in NAWM/NWM and in WM lesions. Care was taken that all areas had the same size which was 80 voxels. The average SMT‐ and DTI‐measures for these ROIs were then calculated. Quantities on 96 ROIS were collected in NWM, MAWM and WM lesions.

### Statistical analyses

Differences in age and sex between patients and HCs were assessed using t‐test and Chi‐square test, respectively. Generalized linear mixed models for binary outcomes were used to assess differences in each of the MRI metrics between different tissues types. In these models, the type of MR measurement (*V*_ax_, *D*_ax_, and AD) was treated as a fixed‐effect factor, and a nested random effect factor for ROI and patient ID was included to take into consideration the data correlation structure. Adjusted odds ratios (OR) with corresponding 95% confidence interval (CI) and the Akaike information criterion (AIC) values were reported. Some of the original values were very small, which resulted in extreme OR values. Therefore, each variable was scaled to the degree needed to have easy‐to‐interpret OR ranges. Specifically, *V*_ax_, *D*_ax_, and AD were scaled 10 (*V*_ax_) and 10,000 (*D*_ax_ and AD) times in all generalized linear mixed models. Associations among different MRI measures and between MRI and clinical variables were assessed using a Spearman's correlation.

Interscan variability in *V*_ax_, *D*_ax_ and AD was assessed using Spearman's correlation analyses, intra‐class correlation analysis and linear mixed‐effect models. These models estimated differences between the two measurements taken 2 weeks apart while adjusting for the fixed‐effect factors, for example, scan (first/second) and ROI (type). The nested random effect factors were subjects and scan. MRI measurements from the two scans were visualized with smoothed scatter plots. Spearman's correlation coefficients were calculated between the two scans.

## Results

All recruited participants tolerated the entire brain MRI scan and there were no missing data.

### Interscan variability (Fig. 3)

**Figure 3:**
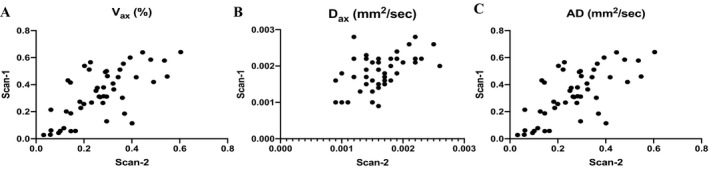
Correlation plots of scan‐1 and scan‐2 values. Correlation plots showing the associations between scan‐1 and scan‐2 measurements of *V*_ax_ (A), *D*_ax_ (B), and AD (C).

As represented in Figure 3, Spearman's correlation analyses showed that scan‐1 and scan‐2 measurements were highly correlated (*r* = 0.654, *P* < 0.0001 for *V*_ax_, *r* = 0.367, *P* = 0.010 for *D*_ax_, and *r* = 0.809, *P* < 0.0001 for AD). Linear mixed‐effect models showed that *V*_ax_ (*P* = 0.337), *D*_ax_ (*P* = 0.326) and AD (*P* = 0.966) values were consistently similar between the two scans. Table 2 reports intraclass correlation coefficients and 95% CI of each pair of measurements.

**Table 2:** Measures of interscan variability

Variable	Intraclass correlation coefficient (ICC)	95% Confidence interval of ICC
*V*_ax_	0.424	0.004/0.483
*D*_ax_	0.300	0.074/0.675
AD	0.275	−0.085/0.637

### Analysis of cBHs vs T2‐lesions (Fig. 4)

**Figure 4:**
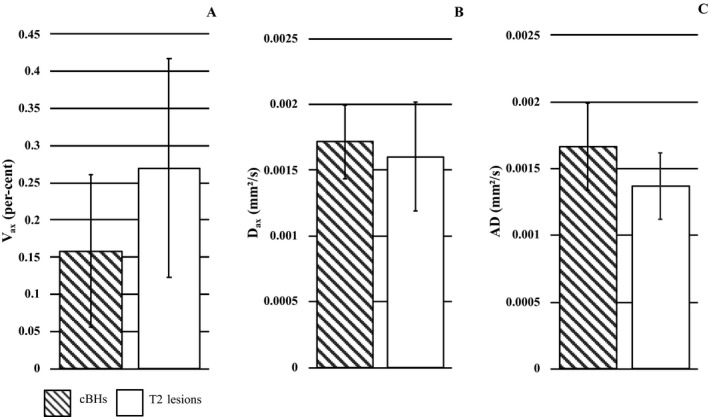
cBHs and T2‐lesions SMT‐metrics. *V*_ax_ (A), *D*_ax_ (B), and AD (C) values in cBHs (patterned columns) and T2‐lesions (white columns). Data are expressed in mean (columns) + 1 standard deviation (bars).

This analysis was obtained in 209 cBHs which were compared with 209 contralateral T2‐lesions identified from nine patients. *V*_ax_ [*P* < 0.0001, AIC = 446.5, OR = 6.25, 95% CI = (4.28, 9.52)] values were consistently lower in cBHs compared with T2‐lesions. *D*_ax_ [*P* = 0.0016, AIC = 571.2, OR = 0.85, 95% CI = (0.77, 0.94)] and AD [*P* < 0.0001, AIC = 482.4, OR = 0.68, 95% CI = (0.61, 0.74)] values were higher in cBHs compared with T2‐lesions. *V*_ax_ values decreased by 63.4 ± 45.5% in cBHs vs T2‐lesions, whereas *D*_ax_ and AD values increased by 3.6 ± 7.9% and 14.1 ± 9.1%, respectively.

In cBHs, only *V*_ax_ and AD measures were significantly associated (*r* = −0.750, *P* = 0.02).

### Analysis of T2‐lesions vs NAWM (Fig. 5)

**Figure 5:**
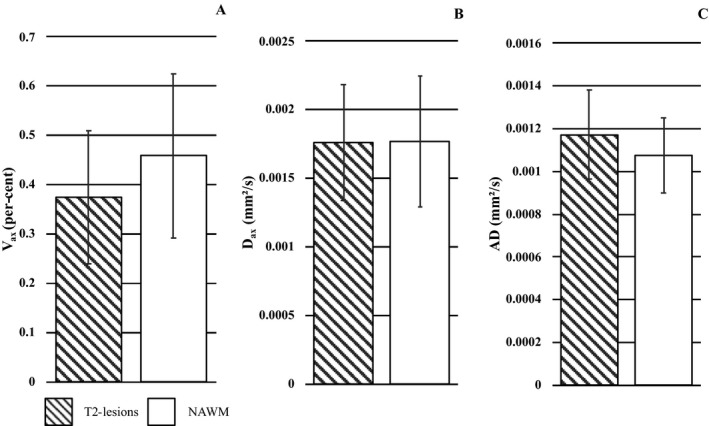
T2‐lesions and NAWM SMT‐metrics. *V*_ax_ (A), *D*_ax_ (B), and AD (C) values in T2‐lesions (patterned columns) and NAWM (white columns). Data are expressed in mean (columns) + 1 standard deviation (bars).

This analysis was obtained in 606 T2‐lesions which were compared with 606 contralateral ROIs identified from 18 patients. *V*_ax_ [*P* < 0.0001, AIC = 1566.9, OR = 0.54, 95% CI = (0.47, 0.61)] values were lower and AD [*P* < 0.0001, AIC = 1614.5, OR = 1.31, 95% CI =  (1.23, 1.41)] values higher in T2‐lesions compared with NAWM ROIs. *V*_ax_ values decreased by 36.5 ± 9.7%, whereas AD values increased by 8.8 ± 0.5% in T2‐lesions compared with NAWM. *D*_ax_ was 3.0 ± 9.4% higher in T2‐lesions compared with NAWM ROIs. This increment, though, did not correspond with significantly different values between the two tissues types.

In this subset of lesions, *D*_ax_ values were significantly associated with *V*_ax_ (*r* = 0.824, *P* < 0.0001) and AD values (*r* = 0.570, *P* = 0.014).

### Analysis of NAWM vs NWM (Fig. 6)

**Figure 6:**
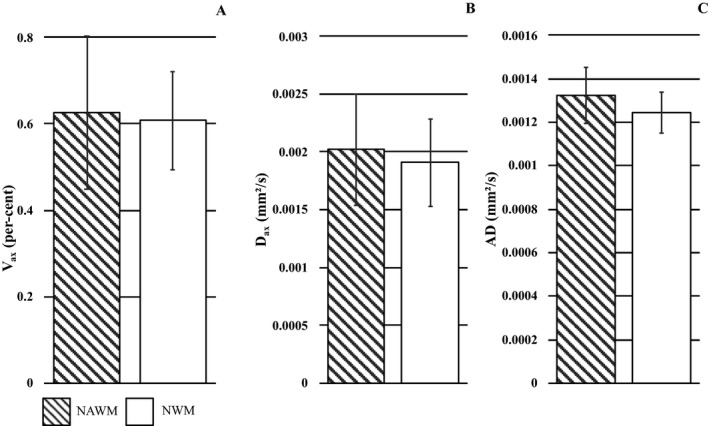
NAWM and NWM SMT‐metrics. *V*_ax_ (A), *D*_ax_ (B), and AD (C) values in NAWM (patterned columns) and NWM (white columns). Data are expressed in mean (columns) + 1 standard deviation (bars).

This analysis was obtained in 136 NAWM ROIs which were compared with 72 NWM ROIs identified from 18 patients and all nine HCs.

To ensure proper anatomical correspondence, comparisons between NAWM and NWM regions was limited to areas of the internal capsula. No group‐differences were seen in *V*_ax_ [*P* = 0.961, AIC = 35.5, OR = 0.95, 95% CI = (0.054, 47.25)], *D*_ax_ [*P* = 0.915, AIC = 35.5, OR = 0.96, 95% CI = (0.30, 4.00)] and AD [*P* = 0.801, AIC = 35.4, OR = 0.68, 95% CI = (0.0056, 10.50)]. These nonsignificant differences corresponded to percent drops of 2.9% in *V*_ax_, and 5.4% and 5.8% increases in *D*_ax_ and AD between NAWM and NWM. NAWM *V*_ax_ was significantly correlated with NAWM *D*_ax_ (*r* = 0.914, *P* < 0.0001).

### Associations between conventional radiological measures and *V*_ax_/*D*_ax_ measurements

This explorative analysis was limited to only the patients who contributed to the measurements, that is, nine patients for cBHs *D*_ax_ and *V*_ax_ and 18 patients for T2‐lesions and NAWM ROIs. Significant correlations emerged between cBHs *D*_ax_ and cBHs volume (*r* = −0.826, *P* = 0.006). None of the diffusion‐based metrics measured in T2‐lesions and NAWM correlated significantly with cBHs and T2‐lesion volume. Statistical trends were seen in the associations between T2‐lesion volume and *D*_ax_ measured in T2‐lesions (*r* = −0.428, *P* = 0.076) and between NAWM AD and cBHs volume (*r* = −0.426, *P* = 0.088).

### Associations between clinical measures and *V*_ax_/*D*_ax_ measurements

As seen for the associations with radiological metrics, this explorative analysis was limited to only the patients who contributed to the measurements. Nine patients for cBHs *D*_ax_ and *V*_ax_ and 18 patients for T2‐lesions and NAWM ROIs. We did not find any significant association between MRI and clinical metrics. A trend was seen only between T2‐lesion *V*_ax_ (*r* = −0.424, *P* = 0.079) and T25‐WT scores.

## Discussion

We deliver the first clinical application of SMT as an imaging tool to probe axonal integrity in brains of patients with MS and show the feasibility of our technique for an in vivo marker of axonal damage. SMT produces two novel metrics namely *V*_ax_ and *D*_ax_. Although changes in *V*_ax_ and *D*_ax_ follow the same directions of those seen with AD, SMT offers a novel framework in that while AD depends on the diffusivity parallel to the axons and their orientation distribution, SMT fully factors out the unwanted effect of the axon orientation distribution (e.g., fiber crossings and intrabundle orientation dispersion) and provides estimates of axonal configuration, only. The removal of orientation dependency increases the specificity of the measurement to axonal integrity, which is fundamental to properly assess disease in human brains.

This translates into the delivery of a more accurate information regarding the degree of injury of myelinated axons. The latter is important information for patients with MS since axonal injury is a key element of their physical and cognitive disability and represents an end‐stage, irreversible disease trait. Treatment strategies targeting axonal counts are much needed to arrest MS progression and an imaging biomarker accurate proxy of this count is fundamental to assess drugs effect.

*V*_ax_, *D*_ax_, and AD values were found to be consistently different between cBHs and T2‐lesions; statistical analyses yielded comparable AIC, hence proving that from a statistical standpoint, the degrees of significance were comparable. However, in cBHs *V*_ax_ values decreased by about 63%, whereas *D*_ax_ and AD values increased only by about 4% and 14%, respectively. The magnitude of these differences points toward a different level of sensitivity to pathology. While *V*_ax_ provides indirect estimates of the apparent axonal volume fraction, *D*_ax_ and AD are also more sensitive to the overall lesion, intra‐ and extra‐axonal, microenvironment where several factors may modulate measurements.

cBHs are known to harbor a diminished number of axons,^4^ whereby the empty space left by axonal loss is filled with cerebrospinal fluid (CSF).^8^ In cBHs axonal death is for the most part secondary to myelin injury, thus axonal loss is nearly always accompanied by myelin death. In this pathological scenario, we argue that increases in AD capture the degree of CSF quantity outside residual axons but also water which accumulates inside axons when swelling proceeds death; decreases in *V*_ax_, instead, are more reflective of viable remaining intact (myelinated) axons, irrespective of their sizes or the surrounding CSF. Differences in the degree of associations between *V*_ax_ and AD or *D*_ax_ values may also be intrinsic to the biophysics of these parameters. AD is sensitive to both orientation and microstructural effects, whereas *D*_ax_ is only sensitive to microstructural features as the orientation distribution effect is factored out by the model. It is likely that in cBHs modifications of the orientation distribution play a larger role compared with that of microstructural changes due to the actual tissue loss and water accumulation, thus rendering AD (more than *D*_ax_) reflective of *V*_ax_ values.

Analyses related to T2‐lesions in comparisons to NAWM were obtained in a larger number of ROIs and patients since virtually every patient presented with lesions on T2‐w MRIs. As seen for cBHs, *V*_ax_ values were lower and AD values higher in T2‐lesions compared with NAWM ROIs, and AICs were comparable. *V*_ax_ values decreased by 36%, whereas AD values increased by 9% in T2‐lesions compared with NAWM. *D*_ax_ measures were minimally higher in lesions compared with NAWM but this difference did not reach statistical significance. *D*_ax_ was significantly associated with T2‐lesions *V*_ax_ and AD. Such associations differ from that seen in cBHs in that *V*_ax_ and *D*_ax_ were positively correlated.

Three possible interpretations, not mutually exclusive, can explain the findings of *V*_ax_ reduction in T2‐lesions. First, our data suggest that axonal injury is not a feature of cBHs only and that, although to a less extent, chronic T2‐lesions may harbor some degree of axonal death. Second, one shall keep in mind that *V*_ax_ may also be affected by myelin loss not necessarily mirrored by axonal death. Myelin degradation makes the tissue substrate less densely packed and may lead to an increase of exchange between intra‐ and extra‐axonal water, which the SMT model assumes as negligible. Untangling the effect of axonal and myelin pathology is difficult in T2‐lesions where: (1) the pathological features are more heterogeneous than in cBHs; (2) most of the axons are expected to be viable; (3) remyelination and demyelination may be tightly spatially connected. This may explain also the complex and intriguing results derived from correlation analysis. Last, in the presence of chronic inflammation and associated free water content changes in the extracellular space, the T2 relaxation time and diffusion signal coming from the extracellular compartment increase. In SMT, the higher intensity of the signal from the extracellular space will translate into a higher signal fraction (1 − *V*_ax_) or lower *V*_ax_. In the absence of compartmental T2 relaxation time data, the determination of this effect remains elusive.

We argue that the pathological heterogeneity of T2‐lesions explains the similarity in *D*_ax_ values between these lesions and NAWM as well as the positive associations between *V*_ax_ and *D*_ax_ values.

Different histopathological stages, macroscopically characterized by changes in axonal size, feature the pathway toward atrophy and consequential death.^27^ Chronic axonal injury is characterized by the accumulation of axonal proteins in the proximal stump and end bulbs of spheroids containing cytoskeletal phosphorylated proteins, such as phosphorylated neurofilament. Axons at this stage are unusually thick due to chronic axonal reaction to demyelination. When the damage is secondary to chronic demyelination, axons tend to swell, accumulate nonphosphorylated neurofilaments and lose phosphorylated neurofilaments. These changes in phosphorylation are consequences of chronic demyelination. Atrophy is a further‐late common‐end stage phenomenon, characterized by decreased axonal volume and thickness and disturbance of neuronal protein synthesis.^27^

It is well‐known that all these processes, along with demyelination alone, re‐myelination, and gliosis, may co‐exist in T2‐lesions. Imaging cannot distinguish any of these pathologies but can indirectly proxy all of them by depicting changes in microstructural integrity (changes in protein content), water content (swelling), and overall axonal quantity (axonal death). All these changes alter water diffusion microenvironment and eventually alter the diffusion MRI signals in potentially opposite directions. Thus, lack of end changes in *D*_ax_ and the positive, in a way “paradoxical” associations seen between *V*_ax_ and *D*_ax_ may be the result of these heterogeneous pathological processes along with sensitivity of *V*_ax_ to both axonal and myelin density when axonal loss has not occurred.

Differences between NAWM and NWM *V*_ax_, *D*_ax_, and AD led to negative results though a strong trend was seen for *V*_ax_. Several factors, not necessarily mutually exclusive may explain our data. First NAWM axonal pathology may not necessarily be present in all MS patients,^28^ especially if one considers that (1) about 50% of patients included in our study had minimal disability EDSS ≤1.0 and (2) that our study targeted only a selected group of WM fibers. Second, it is expected that the magnitude of the differences between NAWM/NWM is smaller than the ones measurable between different lesion types or between lesions and NAWM, thus it is possible that our sample size was not powered to detect them. Third, opposite to as seen in lesions, axonal injury in NAWM is not secondary to demyelination rather to Wallerian (primary) axonal degeneration which eventually leads to secondary demyelination. Thus, several injured axons may still be myelinated, a factor that may prevent significant changes in diffusion‐based parameters to be measured.

Notwithstanding the above considerations, it is interesting to note that *V*_ax_ measurements were nearly significantly different between the two groups of ROIs, whereas AD and *D*_ax_ were not. The data indicate once again the ability of *V*_ax_ to depict pathologies that are different and are likely measurable earlier in the disease course than those captured from *D*_ax_ and AD.

Of all explored associations, the only one that showed a statistically relevant (inverse) significance was that between cBHs *D*_ax_ values and cBHs volume. In lack of other data corroborating the biological meaning of these findings, any interpretation remains limited and vulnerable. It is important to underscore that SMT metrics were derived only from a limited number of patients and lesions. On the contrary, volume measures reflect the overall lesion burden each individual patient had. This factor, in addition to clinical heterogeneity of the study cohort and its relatively small size make it not suitable to answer specific biological‐clinical questions.

### Study limitations and conclusions

As stated, our work lacks data regarding clinical impact of SMT‐derived metrics. While we acknowledge that this is an important limitation, we also point out that this line of research was outside the scope of our work. Assessing the ability of SMT to quantify tissue changes was our study goal and it is the first important pre‐requisite for a given sequence to be further applied on larger clinical scale. Our study is part of a larger project the goal of which is assessing the role of SMT‐derived metrics as hallmarks of neurodegenerative tissue injury and severity of neurological dysfunction.

We also lack analysis of active lesions, featured by contrast enhancement and overt blood–brain barrier breakdown. This topic was also outside our initial scope of proving feasibility of SMT in patients with MS. It is the focus of future work as very important from both a biological and a technical stand point.

Another important limitation is that, except for myelin water that is invisible at the echo times used in clinical diffusion MRI scans, the SMT model assumes uniform T2 values (i.e., the same value for intra‐ and extra‐axonal spaces). Although this assumption has been used extensively thus far, a recent study^29^ reported that the intra‐axonal T2 is longer than the extra‐axonal T2, which may result into a biased estimation of the apparent *V*_ax_ obtained using SMT. The same study also found that the intra‐axonal T2 could be dependent on the angle of the tract relative to the main magnetic field, which suggests that subject's head orientation itself could influence the results. A possible solution is to perform combined diffusion‐relaxometry measurements. In terms of clinical translation, though, one needs to be cautious as acquiring T2‐maps adds another dimension of acquisition and significantly increases the total scan time. By contrast, SMT provides a simplified and efficient means to evaluate the apparent axonal volume fraction with clinically feasible scan time, if one is mindful of the caveat that absolute values may be confounded by the multi‐compartment T2 relaxation.

In conclusion, we propose the first clinical application of SMT in brain of patients with MS. Our results yield intriguing observations which overall testify for the applicability of this technique in patients with brain and spinal cord^16^ conditions featured by neurodegeneration. Our group is committed to future work to resolve some of the issues related to the complexity of the technique and its findings. Notwithstanding the discussed limitations of our study, we offer a solid demonstration that SMT provides a conceptually novel framework to look at axonal pathology in MS patients and represent a step forward in the in vivo characterization of MS pathology.

## Conflict of Interest

None declared.
